# The Clergy

**Published:** 1889-06-15

**Authors:** 


### The Hospital

A Weekly Institutional Journal of

**Science, Medicine, Nursing, and Philanthropy.**

*For Hospitals, Asylums, Medical Practitioners, Students, Nurses, Families, Parishes, Congregations, and the Charitable Public.*

Vol. VI. No. 142.] [June 15, 1889.

### The Clergy.

Is the hot summer season the best time for the Hospital Sunday collection, or would not the mind-bracing atmosphere of winter be more conducive to a successful result? That raises another question, viz., whether the quality of the Hospital Sunday sermon really has much to do with the amount collected? For our part we think it has; and it is probable that if the facts could be fully known, it would be found that the clergyman or minister who most thoroughly prepares himself to deal with the subject of hospitals, their merits and their needs, will, other things being equal, invariably influence in a favourable direction the minds and purses of his hearers. But is the collection of the day the sole or even the principal object which an intelligent and far-seeing preacher will aim at in his Hospital Sunday sermon? It is the chief function of the clergy to influence the minds of their congregations in favour of intelligent voluntary action. Every religion recognises the difference between willing obedience and mechanical constraint. The State represents authority and compulsion, and succeeds in extracting from its subjects a certain kind of conformity. The Church, all Churches, represent teaching, persuasion, the improvement of mind, character and conduct by means of enlightened intelligence and convinced judgment. The origin and growth of voluntary hospitals are direct results of the gradual working into the people's minds of the religious and humane ideas of the Old and New Testaments, by means of the public and private teaching of the clergy. At the present moment the preacher, when he makes his appeal for hospitals, knows that he casts his seed into prepared ground; and he anticipates his crop with the same certainty as the farmer who sows his wheat in October or his barley in March.

But hospitals, like agriculture, have fallen on bad times; and just as the intelligent landlord and capable farmer are casting about to discover, if possible, new methods of making their business profitable, so, it seems to us, should the clergy consider whether or not it is possible that a higher and more productive level of good works may not be attained by their congregations in the direction of hospital support. Let no cynically minded person suppose we are looking at the question merely from the point of view of the hospital manager who, so long as the funds come in, cares little how they are obtained. We are at least making an effort to place the hospital question on the high level of devoted religious work. Here is a great cause, the cause, first of the poor, and then of the sick poor. Strip it of every adventitious circumstance of every kind, it is still so humane and so worthy in itself as to deserve to be placed in the first rank either of religious or of secular good work. It appeals to the highest principles of the man who strives to do all things "for the glory of God," and to the deepest instincts of him who steadfastly aims at what is best "for the love of man."

Admitting, as everyone does, the greatness and worthiness of the cause of hospitals, can it be fairly considered that the religious people of the metropolis display that ardour and that generosity which it would be reasonable to expect? And if they do not, are the clergy of the Church of England, and other ministers of religion, in any way responsible for the dead level of the last ten years? For it cannot be denied that the last ten years have seen so insignificant a rise of enthusiasm that it is hardly worth calling a rise at all. Is it true that, with a few rare exceptions, the preachers who advocate hospitals on Hospital Sunday have little knowledge of the subject, and less regard for it? Is it true that their appeals consist largely of mere frothy attempts at sentiment, without any depth of conviction behind them? We do not like to select particular instances; but two churches occur to the mind, both of which consist, to a large extent, of prosperous people, and both of which raise annually for all purposes probably about the same large total. Yet one of these two churches last year collected more than a thousand pounds for hospitals, whilst the other was content to give little more than a hundred.

Now, giving to hospitals is either a religious duty or it is not. If it be, then he who gives should give with all his heart. That duty is incumbent on the preacher first of all. He may not be called upon to give much money, for he may not have it to give; but he is called upon to give more than money. He gives, or should give, intelligent instruction, reasoned convictions, motive, incitement; it is for him, in fact, to raise the act of giving to hospitals to the highest level of a sacred duty—a duty which is not done at all unless it be done with the whole heart, and to the utmost extent of each individual giver's power. But does it not strike everyone who thinks about the subject, that the Hospital Sunday collection has fallen into the rut of mechanical routine, and has hardly any flavour of religious duty about it at all? Some forty thousand pounds are raised every year whether much or little effort be made; whether there be the previous preparation of a Hospitals' Week or not. Does this afford any evidence whatever that either the gifts or the givers are inspired by religious enthusiasm? Religious enthusiasm has its ebbs and its flows, its depths and its heights, its music and its silence. It is but too plain that Hospital Sunday has taken little hold upon religious sentiment and conviction, and only affects the people by the force of custom and mechanical routine.

Ought the clergy to be satisfied with this condition of things? The mere monetary result concerns hospital managers; but a much higher question concerns the teachers of religion. If their hearers are deaf to appeals which everybody admits, the presumption is that they are deaf to other appeals which carry no such general authority of conviction with them. If the hand be not prompt to the performance of manifest religious duty, it is practically certain that the heart is alienated from deep religious earnestness. Truth to say, the leaders of the philanthropic movements of the times are not happy at the prospect that lies immediately before them. They see no evidences of elasticity in the public sentiment with regard to hospitals; no more readiness or warmth of response to their appeals in the churches than in the world outside. If religion, the Christian religion, fails the hospitals, what is to become of the poor and the sick? It is, perhaps, not fair to throw upon the clergy the whole weight of the responsibility which attaches to the religious and philanthropic indifference of the times, but if any enthusiasm is to be kindled in the congregations it would seem that it must begin in the pulpit.

